# Family Members' Needs in Internal Medicine Wards and the ICU: A Comparison

**DOI:** 10.1111/jep.70239

**Published:** 2025-08-19

**Authors:** Tatyana Lupyan, Revital Zelker, Shadi Abomokh, Adena Brickman, Sigal Shafran‐Tikva, Rely Alon, Muriel Cohen, Julie Benbenishty

**Affiliations:** ^1^ Hadassah Hebrew University Medical Center Jerusalem Israel; ^2^ School of Nursing Jerusalem College of Technology, Hadassah Hebrew University Medical Center Jerusalem Israel

**Keywords:** family needs, hope, ICU, internal medicine ward, mechanical ventilation, reassurance, threat

## Abstract

**Rationale:**

Families of patients being mechanically ventilated in hospital ward or ICU settings, may experience significant morbidities. A comparison of the needs in these two populations is necessary to address the challenges they experience.

**Aim:**

Explore the needs of families of ventilated patients located in the ICU compared to those whose family member is being ventilated in an internal medicine ward.

**Methods:**

This was a cross‐sectional secondary analysis of data collected between 2013 and 2019 in ICU and internal medicine departments. Participants in both settings were aged over 18 years, gave their consent to participate in the questionnaire‐based study, and were either family or legal relatives of a hospitalised patient. Inclusion criteria required a hospital length of stay of more than 2 days.

**Results:**

A total of 221 family members were recruited: 89 from the ICU and 132 from internal medicine departments. The results indicated that families of a patient in an internal medicine ward required significantly more support and proximity than those with a family member in the ICU. In contrast, the ICU associated families demonstrated significantly higher needs for information than those with a family member in an internal medicine ward.

**Conclusion:**

Families of ventilated patients in internal medicine wards and ICUs have unfulfilled needs that should be considered. Different clinical settings present diverse challenges, which clinicians need to know how to address. The climate and nature of the clinical ward or unit influence the family's needs.

## 1 Introduction

Hospitalisation is a harrowing experience for both the patient and their family, and the problems become even more complex if the patient requires life‐saving mechanical ventilation (MV). The Israeli Ministry of Health reports an increase in the number of ventilated patients admitted to hospitals [1]. Some of these patients may need ICU facilities, but are hospitalised in other units, mainly in internal medicine wards due to the lack of intensive care beds [2].

Caring for MV patients requires extensive knowledge and unique skills. Similarly the family members of such patients also have special needs. The needs of a family with a family member hospitalised in the ICU have been well described [3, 4]. However, there is little to no information about how such needs are met when the patient is receiving treatment in an internal medicine ward, where the nurse/patient ratio is much lower than in the ICU. The purpose of this study was to compare the needs of families of an MV patient hospitalised in the ICU or in an internal medicine ward.

## 2 Background

Critically ill patients are defined as patients needing intensive monitoring and specialised treatment such as mechanical ventilation [5]. The needs of families with a critically ill family member have been addressed by many countries including Saudi Arabia, Iran, USA, Israel, and Korea [3, 4, 6]. Almost all such studies use the critical care family needs inventory (CCFNI) instrument to assess the family needs [7]. Most investigators agree that the top needs to be met are information and assurance [6, 8, 9, 10, 11].

A 2013 literature review of 30 studies reported that family members considered that these needs were generally unmet in the ICU setting although there was a consensus that nurses were best at attempting to satisfy the family needs [4]. A 2017 integrative review of 16 reports, albeit with varying methodologies, concluded that the needs of families of critically ill patients included; information, making sense, hope, support, involvement, and protection [12].

A 2019 study attempt to develop a model of factors that could assuage the needs of family with a relative in the ICU, identified one demographic variable (family member gender) and three psychological factors of anxiety, depression, and coping self‐efficacy as having a direct and intervening effect on meeting the needs of ICU patient family members [13].

### 2.1 Conceptual Framework

The Virginia Henderson needs theory, one of the most well‐known nursing models, categorises 14 basic human needs. A nurses' role is to act in a substitutive (what a nurse does for the person), supplementary (helping the person), and complementary (working with the person) fashion, with the aim of assisting the individual become as autonomous as possible in obtaining these needs [14]. One of the activities that Henderson considers critically important to a nurse is communication with others in expressing emotions, needs, fears, or opinions [15, 16]. According to Henderson's model, nurses should communicate with family members of a MV patient by using soothing, encouraging tones to explain and describe all the devices attached to the patient and should assess family needs, while providing care for the ventilated patient. Nurses should be explicit and use a simple understandable vocabulary when communicating with the family.

At the triage stage of hospitalisation, some critically ill patients may be judged as unlikely to benefit from intensive care (frequently because of disease acuity and high predicted mortality) and may be admitted to a more general ward. Often, the criteria that determine which patients are most likely to benefit from the ICU are not evidence based [17] and the most acutely ill patients are not admitted to ICU. This is mainly applicable to elderly patients with multiple chronic illnesses [17].

There has been little research concerning families of ventilated patients hospitalised in internal medicine wards. It might be assumed that these families have the same needs as family members of ICU patients. However, the ICU climate, as well as the level of education of ICU advanced specialised trained nurses are very different from the situation in internal wards and may not meet the needs of families of mechanically ventilated patients hospitalised in internal medicine wards.

A retrospective analysis published over two decades ago in Israel revealed an increase in overall ventilator‐days from 11,164 (31 patients/day) in 1997, to 24,317 (67 patients/day) in 2016. This significant growth was attributed mainly due to the presence of more ventilated patients in internal medicine wards (1997: 4 patients/day; 2016: 24 patients/day) [1, 18]. This may reflect the insufficient number of ICU beds in Israel. A previously published detailed snapshot (over 4 months) of patients ventilated on internal medicine wards in Israel (*n* = 745) revealed that the patients tend to be elderly (median age 75 years) and that 24% were ventilated for more than a week. Notably, prolonged mechanical ventilation may have specific effects on family members' needs, for example an increase in financial burden. Caregivers also reported long‐term physical and psychological morbidity and health changes including alterations to sleep, energy, nutrition, and body weight [19]. Hospital‐wide ventilation patterns calculated as the weighted sum of the various individual patient units revealed a winter peak in the internal medical wards and in the emergency department. This seasonality is not surprising, given the greater incidence of winter respiratory ailments [18].

The needs of family members of mechanically ventilated patients have been well addressed by interventional research methodology [20]. However less is known regarding family needs in other clinical environments, specifically in internal medicine wards.

A Canadian qualitative study reported that ICU family caregivers received inadequate informational, emotional, training, and appraisal support from health care teams. The interviewees revealed a limited understanding of the implications of prolonged ventilation and did not participate in decision‐making processes regarding readiness for weaning.

The aim of this study was to explore and compare the needs of family members of MV patients in the ICU compared to those ventilated in internal medicine wards.

## 3 Methods and Design

This study comprised a secondary analysis of two studies conducted at the same medical centre, a large tertiary hospital located in West Jerusalem, Israel. The first was an interventional study designed to evaluate the contribution of family support group for family members with a relative hospitalised in the ICU. The purpose was to explore the impact of a nurse led support group on families of ICU patients. Initially we preformed a qualitative analysis of themes determined during the group meetings [3] Secondly, we used a quantitative design we aimed at investigating if the support group fulfilled family's need. We asked if their participation in the support group fulfilled each need in the questionnaire [11].

The other study explored the needs of family members of MV patients in four internal medicine wards. We used self‐reporting questionnaires that were distributed to first‐degree relatives or spouses/adoptive family members. This paper reports a comparison cross‐sectional secondary data analysis and therefore required no additional ethical approval.

### 3.1 Ethical Considerations

The original studies received Institutional approval (IRB approvals ICU study: 0329‐13, internal medicine study − 0477‐16).

#### 3.1.1 The ICU Setting

The ICU setting was a 14 bed ICU with a nurse/patient (N/P) ratio of 1/2, where most of the nurses possess a specialised post graduate clinical specialty ICU competency training certification. The ICU environment has a private room for each patient with windows, and is a closed unit with specific visiting hours. The family waiting room is a very large area close to, but outside, the ICU, which is equipped with a full kitchen, scenic view, and comfortable seating. Visiting hours are three times a day for 1 h each visit.

#### 3.1.2 The Internal Medicine Setting

The internal medicine setting comprised four internal wards, each with 36 beds. All the internal medicine departments have one room that functions as a step down/high dependency unit for patients needing higher acuity care. This room has an N\P ratio of 1\4 compared to the usual N\P ratio of 1/8. Ventilated patients may be hospitalised either in regular rooms throughout the ward or in the high dependency unit. Regular rooms hospitalise up to three patients with a dedicated bed for relatives or caregivers. The regular rooms are open to visitors 24 h/day, with the high dependency unit open to visitors 16 h/day, not including 8 h at night.

### 3.2 Participants

Participants in both settings were aged over 18 years, gave their consent to participate in the study, and were either family or legal relatives of the hospitalised patient. The inclusion criterion was a hospital stay of more than 2 days. Participants were informed that participation in the study was voluntary, and that ethical approval had been received from the Institutional review board of the hospital. The secondary analysis used a database of 221 family members of whom 89 were caregivers in the ICU and 132 in internal medicine wards.

### 3.3 Study Sample

the ICU sampling was a convenience sample, while the internal medicine wards study sample was based on the assessment of an average of six ventilated patients per day. Calculations with a significance level of 95%, power of 80%, confidence interval of 0.05, and response rate of 50%, yielded a minimum sample size of 132 family members.

### 3.4 Data Collection

A family member identified as fulfilling the inclusion criteria was approached by a study nurse who then provided details about the study. After consenting, the family members completed the critical care family needs inventory tool (CCFNI). The ICU family questionnaires were collected between March 2013 and December 2014, while the internal medicine data collection period was from January 2016 until December 2019, due to fewer turnover of MV patients in internal medicine compared to ICU.

#### 3.4.1 Data Collected Using

Data collected using the CCFNI questionnaire relates to 45 needs that participants rated on a scale from 1 (not important) to 4 (very important). The questions are divided into five dimensions: assurance, proximity, information, comfort, and support [7].

Demographic data collected included gender, age, ethnicity, and relationship to the patient. The CCFNI, which was written in English, was translated to Hebrew by two independent bilingual Hebrew/English speakers. This output was then back‐translated by two other independent bilingual translators to verify the final version [21].

#### 3.4.2 Rigour

The psychometric properties of the original version of the CCFNI were assessed in 1991, and yielded a reliability of α = 0.92 for the general scale [22]. The five dimensions of the original version of the CCFNI had an acceptable level of reliability with a reported internal consistency: assurance (α = 0.61), support (α = 0.88), proximity (α = 0.71), comfort (α = 0.75), and information (α = 0.78) [7, 22].

In the current study, internal consistency was tested after the tool was translated into Hebrew.

The Cronbach alpha scores for the five subscales were:

- The ICU study ‐ Assurance (α = 0.69); support' (α = 0.78); proximity (α = 0.65); comfort (α = 0.80); information (α = 0.75).
- The internal medicine wards study ‐ Assurance (α = 0.78); support (α = 0.83); proximity (α = 0.57); comfort (α = 0.55); information (α = 0.68).

#### 3.4.3 Data Analysis

Frequencies and proportions of the demographic data were calculated by the SPSS (version23) statistical package. We have previously (2019) merged the databases of the ICU and internal medicine wards studies into a single database to facilitate data analysis. The CCFNI item scores were compared to assess differences and similarities between the two studies. The mean scores for each item were compared using *t*‐tests. Any significant differences between the individual items were also assessed by *t*‐tests.

## 4 Results

A total of 221 family members: 89 ICU caregivers and 132 from internal medicine departments were included in the study. As presented in Table 1, the average age was between 49.3 and 51.80 years old, the majority were female, and the average length of current hospitalisation in the ICU/wards was 6 days. The only significant difference was that 50% of the family members caring for patients in the ICU were over 60 years old compared to 32% over the age of 60 in internal medicine wards.

**TABLE 1:** Gender, age and ethnicity of family members.

Demographic variables	ICU (*n* = 89)	Internal medicine ward (*n* = 132)
Spouse	61%	75%
Females	60%	63%
Mean age in years (SD)	49.3 (20.6)	51.80 (15.22)
Age over 60	50%	32% * *p* < 0.05
Ethnicity		
Jewish	83%	76%
Arabic	17%	24%
Length of hospitalisation (days) mean	6.2 days	6.1 days

**FIGURE 1:**
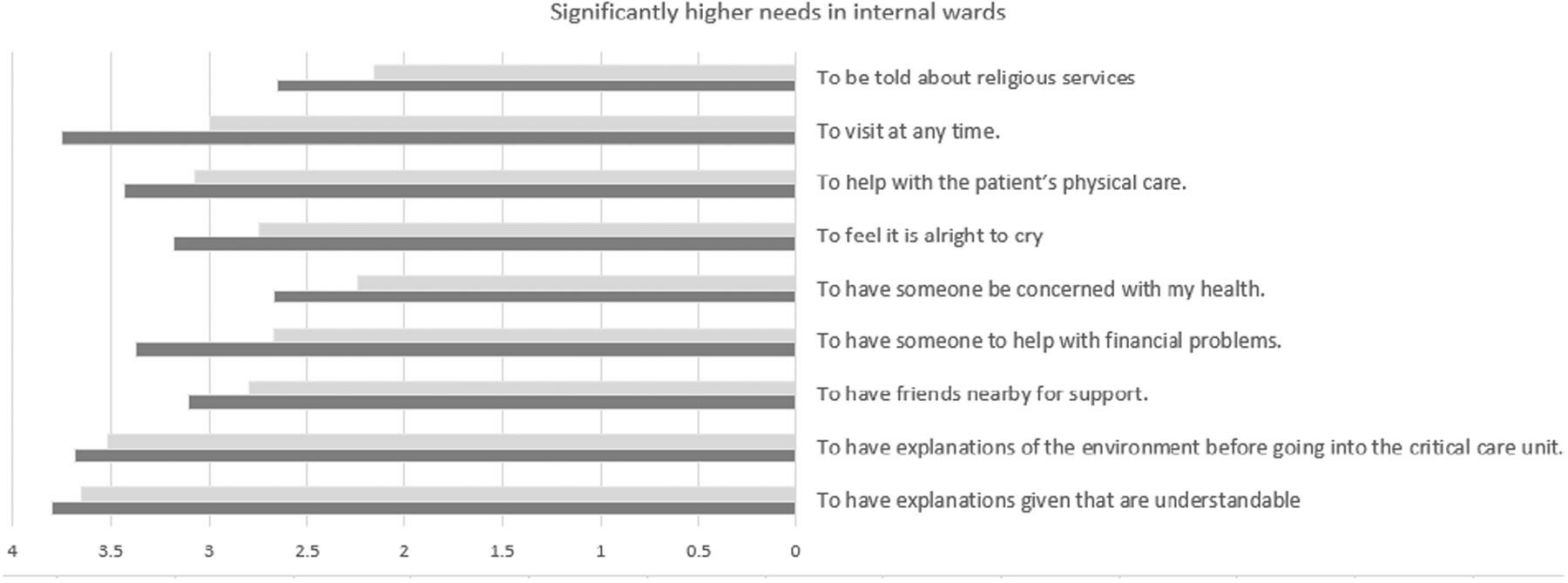
Significantly higher needs in internal medicine wards compared to the ICU, Dark grey—internal medicine ward family answers, Light grey—ICU family answers.

**FIGURE 2:**
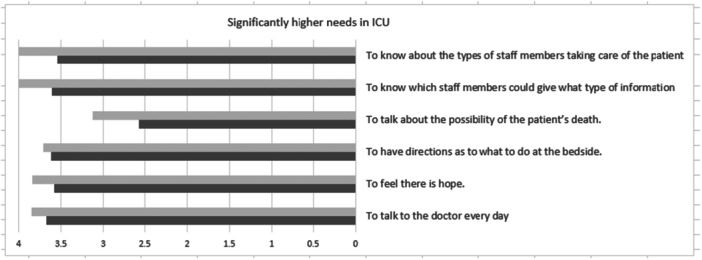
Significantly higher needs in the ICU compared to internal medicine wards, Light grey—ICU family answers, Dark grey—Internal medicine family answers.

CCFNI items is presented in Table 2. The highest score (indicating higher needs) was for assurance, specifically for items dealing with best care provided for their loved ones and feeling that the hospital personnel care about the patient. The lowest scores were in the dimension of support. Items with significant differences are presented inn Figures 1 and 2.

**TABLE 2:** Comparison between ICU and internal wards family member needs scale—mean, SD and *t* test.

CCFNI item	Internal ward (*n* = 132) Average score	ICU (*n* = 89) Average score	*p* value
**Assurance**	3.81 (0.55)	3.84 (0.43)	0.36
To know the expected outcome.	3.78 (0.55)	3.78 (0.62)	0.93
To talk to the doctor every day.	3.67 (0.71)	3.85 (0.35)	0.01
To have questions answered honestly.	3.88 (0.42)	3.87 (0.32)	0.87
To feel there is hope.	3.58 (0.86)	3.84 (0.42)	0.004
To be assured that the best care possible is being given to the patient.	3.96 (0.27)	3.93 (0.28)	0.42
To have explanations given that are understandable.	3.80 (0.54)	3.65 (0.61)	0.06
To feel that the hospital personnel care about the patient.	3.93 (0.31)	3.95 (0.21)	0.77
To know specific facts about the patient's progress.	3.90 (0.37)	3.83 (0.36)	0.18
**Support**	2.99 (1.12)	2.78 (1.07)	0.00
To have explanations of the environment before going into the critical care unit for the first time (about MV).	3.68 (0.65)	3.52 (0.56)	0.05
To talk about feelings about what has happened.	2.89 (1.18)	2.76 (0.90)	0.38
To have directions as to what to do at the bedside.	3.62 (0.72)	3.71 (0.79)	0.01
To have friends nearby for support.	3.11 (1.00)	2.80 (0.95)	0.02
To have a place to be alone while in the hospital.	2.86 (1.02)	2.93 (0.97)	0.59
To have someone to help with financial problems.	3.37 (0.94)	2.67 (1.09)	0.00
To have a religious leader visit.	2.51 (1.22)	2.26 (1.10)	0.13
To talk about the possibility of the patient's death.	2.57 (1.23)	3.13 (1.03)	0.001
To have another person with me when visiting the critical care unit.	2.81 (1.09)	2.56 (1.15)	0.12
To have someone be concerned with my health.	2.67 (1.09)	2.24 (1.12)	0.008
To feel it is alright to cry.	3.18 (1.08)	2.75 (1.17)	0.008
To be told about other people that could help with problems.	3.24 (0.95)	3.20 (0.84)	0.79
To be alone at any time.	2.77 (1.17)	2.56 (1.03)	0.18
To be told about religious services.	2.65 (1.23)	2.16 (1.10)	0.003
**Information**	3.66 (070)	3.74 (0.57)	0.01
To have a specific person to call at the hospital when unable to visit.	3.59 (0.75)	3.68 (0.58)	0.32
To know which staff members could give what type of information.	3.61 (0.70)	4 (0)	0.00
To know why things were done for the patient.	3.78 (0.49)	3.76 (0.45)	0.70
To know about the types of staff members taking care of the patient.	3.54 (0.79)	4 (0)	0.00
To know how the patient is being treated medically.	3.85 (0.47)	3.84 (0.39)	0.83
To know exactly what is being done for the patient.	3.86 (0.42)	3.82 (0.40)	0.55
To help with the patient's physical care.	3.43 (0.97)	3.07 (0.97)	0.01
**Proximity**	3.54 (0.86)	3.38 (0.84)	0.00
To visit at any time.	3.75 (0.55)	3.0 (0)	0.00
To talk to the same nurse every day.	2.28 (1.22)	2.32 (1.08)	0.79
To be told about transfer plans while they are being made.	3.81 (0.52)	3.73 (0.54)	0.33
To be called at home about changes in the patient's condition.	3.78 (0.55)	3.68 (0.72)	0.27
To receive information about the patient at least once a day.	3.78 (0.56)	3.83 (0.37)	0.39
To see the patient frequently.	3.84 (0.45)	3.71 (0.59)	0.10

*t*‐tests (2 tailed) of the mean scores in each dimension of the CCFNI revealed significantly higher needs for support and proximity in internal medicine wards compared to the ICU and higher needs for information in the ICU compared to internal medicine wards.

The needs to talk to the doctor daily, to feel there is hope, to be directed what to do at the bedside, and to know which staff members could provide information, scored significantly higher in the ICU compared to the internal medicine families. As a corollary, needs scoring significantly higher in internal medicine family included, having explanations of the environment before going in to see the patient for the first time, having friends nearby for support, having assistance with financial difficulties, being informed about religious services, help with the patient's physical care, being able to visit at any time, to have someone concerned about their health, and the ability to feel it is all right to cry.

## 5 Discussion

The aim of this study was to explore differences in the needs of families caring for a mechanically ventilated patient in the ICU or in internal medicine wards. The most remarkable difference was the significantly higher need for internal medicine families for the company of friends and the ability to visit at any time. This finding was surprising since visiting hours in the ICU are more limited than in internal medicine wards, which are open at least 16 h a day. Hence, the perception of the need to visit is not related to the actual visiting hours policy.

In general, regardless of the place of hospitalisation, the two domains most associated with greater needs were assurance and information. This result is in accordance with previously published findings [4, 6, 8, 9, 10, 11].

### 5.1 Higher Family Needs in ICU Compared to Internal Medicine Wards

The ICU is a high traffic environment that employs a higher ratio of senior and junior physicians than found in internal medicine wards [23]: Medical education and training in Israel). Nursing students rotate through the ICU as part of their intensive care course training, with the result that there are many more available nursing hands to take care of the patients than in an internal medicine ward, where a single nurse might manage the care of four patients in the high dependency unit and up to eight patients in the regular ward. Senior physicians hold a biweekly round in the internal medicine wards in comparison to daily senior physician rounds in the ICU.

Similarly, according to hospital policy, a senior physician speaks daily with family members of patients in the ICU. Because the senior physicians rotate frequently in the ICU, this daily task may be carried out by different physicians, which may impact continuity of care and this may explain the observed increased need for daily information delivered by the doctor, and greater need for assurance expressed by ICU family caregivers. Another possible explanation is that the highly technical environment around the ICU patient induces a greater sense of threat in family caregivers [24], which in turn increases their need for information at the patient's bedside. Family members are often observed staring at the ICU patients' monitor for long periods of time, obsessing about the numbers they see. There is a general perception that whatever their actual condition, their family member in the ICU is in a life‐threatening condition until they are transferred to a regular department [25]. Despite explanations provided by the ICU team, family caregiver might still feel intimidated by the ICU environment [24, 26]. The heightened perception of imminent death in the ICU compared to an internal medicine ward may also explain why families of patients in the ICU express a greater need to be reassured about hope than those whose family member is hospitalised in an internal medicine ward.

ICU hospitalisation of a relative was described as a long and harrowing journey accompanied by emotional chaos, and unstable state of mind as well as a sense of loss of control. This emotional turmoil may be difficult to control and deescalate as the sick relative recovers and is no longer in a life‐threatening condition [26]. Another factor enhancing the feeling of loss of control and uncertainty is the fact that relatives of ICU ventilated and sedated patients may become a substitute decision‐maker during discussions about options for supportive or life‐saving treatment [27].

A Canadian study that examined family presence during ICU rounds revealed that almost 60% of families chose not to attend. Although those who did expressed satisfaction with their involvement, they complained that time restraints prevented their receiving explanations, for example about sensitive test results. Often, the only question addressed to the relative during rounds was whether they had any questions, without leaving enough time for effective quality explanations [28].

One issue highlighted by our findings is that families in ICU need more direction and explanation about who can provide information and about the people caring for their relative. Although all the physicians, nurses, physical therapists, and nutritionists working in the ICU carry a name tag specifying their name and profession, they all wear the same surgical scrubs, with the same colour. In contrast, nurses in the internal medical wards wear a white uniform, junior physicians wear scrubs, and senior physicians have white lab coats. Perhaps the unity of appearance that is unique to the ICU might contribute to the confusion of the family while conversing with either a nurse, physician or other look alike. Literature evidence suggests that information about the patient's medical condition is most frequently provided by doctors and is then frequently reiterated and 'translated' by nurses. Families most often chose doctors to ask about the medical status while enquiries about the equipment, monitors, observations, and consciousness level of the patient are addressed to nurses [29]. Redirection of a question by a physician to a nurse or vice versa frequently adds to the confusion, turmoil and uncertainty ICU families need to cope with [25].

### 5.2 Higher Needs in Internal Medicine Wards (IW) Compared to ICU

The greater need for information expressed by family caregivers in the medical internal wards may be due to the fact that nurses in the ICU are managing the care of two acutely ill patients as compared to the four or eight patients under the care of each nurse in an internal medicine ward. Another contributory factor could be the higher level of training of the ICU nurses. Many Israeli ICU nurses are members in professional ICU nursing association that sets the national policy for care, and provides up to date translational research and regularly scheduled conferences, while Internal medicine nurses do not usually have this level of professional exposure [30]. The greater need for information may also be related to the greater need for emotional support expressed by family caregivers in internal medicine wards. Other contributing factors could be the prolonged period of ventilation in the internal medicine wards as compared to time spent in the ICU and the availability of family support groups in the ICU.

This prolonged time on ventilation, which can impact family economics, may also explain why family carers of patients in internal medicine wards express a higher financial need than those in the ICU.

## 6 Limitations

This was a single centre study, which limits the generalisability of our findings. However, it may serve as the foundation and pilot for further multi‐centre studies. Another point is that the data from the two settings were not collected during the same period: the ICU study in 2013; and the internal medicine wards in 2016. Notably, the instrument chosen to measure family needs was developed using an ICU family population [7]. This is because there is no known tool developed for measuring the needs of family members of MV patients hospitalised in internal medicine wards, but we appreciate that the chosen tool may not be as suitable for this population.

## 7 Conclusions

We believe that the results of this study illustrate the specific support, information and assurance needs of the previously uninvestigated population of families of patients being ventilated in internal medicine wards. More nursing interventions need to be developed and implemented to answer the needs of such family caregivers. The study centre holds weekly support group sessions for families in the ICU, and a similar support group directed by internal medicine nurses could be formed for the families of MV patients hospitalised in the internal medicine wards. We can conclude that more research needs to be done in internal medicine departments about nursing input in particular related to families of MV patients hospitalised in internal medicine.

## Conflicts of Interest

The authors declare no conflicts of interest.

## Data Availability

The data that support the findings of this study are available on request from the corresponding author. The data are not publicly available due to privacy or ethical restrictions.
